# Outcomes of a Reimplanted Talus After a Total Open Extrusion

**DOI:** 10.7759/cureus.9678

**Published:** 2020-08-12

**Authors:** Hichem Issaoui, Mohammed-Reda Fekhaoui, Hatem Abbassi, Mahdi Gargouri, Mazen Ali

**Affiliations:** 1 Department of Orthopedic Surgery and Trauma, Regional Hospital Center of Orleans, Orleans, FRA; 2 Department of Trauma and Orthopedic Surgery, Ibn Sina University Hospital, Faculty of Medicine, Mohammed V University of Rabat, Rabat, MAR

**Keywords:** total traumatic extrusion, open, talus, reimplantation

## Abstract

Total traumatic extrusion of the talus is a severe and disabling ankle injury that requires a high energy trauma. Many treatment options exist and none of them guarantee a successful result. Here, we present the case of a 67-year-old woman who experienced an open total traumatic extrusion of the talus. Based on the principles of open fracture management, we have realized an early administration of antibiotics and tetanus toxoid booster followed by an urgent debridement of the wound. Next, the talus was reimplanted and fixed with a K-wire. This allowed us to avoid the common complication and achieving good clinical outcomes. In our opinion, this is an encouraging and reasonable treatment option unless the talus is grossly contaminated or missing.

## Introduction

Open total traumatic extrusion of the talus is a rare and challenging injury that represents 0.06% of all dislocations and 2% of all talar injuries [1]. It occurs after a high energy trauma with excessive tibiotalar dorsiflexion or plantarflexion combined with subtalar supination or pronation [2]. The talus loses all soft-tissue attachments and its anatomical relationships with tiba, calcaneus and navicular bone [3]. This injury presents a high risk of infection, avascular necrosis, arthritis and modest functional outcome. Until now, there are no consensus about the treatment, and talectomy with primary tibiocalcaneal arthrodesis was the approved treatment [4]. Recently, immediate reimplantation of total extruded talus has been reported in literature with favorable outcomes and has been more recommended as safest and more appropriate procedure [5]. Here, we present the case of a 67-year-old woman who experienced an open total traumatic extrusion of the talus that we treated with an urgent debridement, a talus primary reimplantation and a lesser invasive K-wire fixation. 

## Case presentation

**Figure 1:**
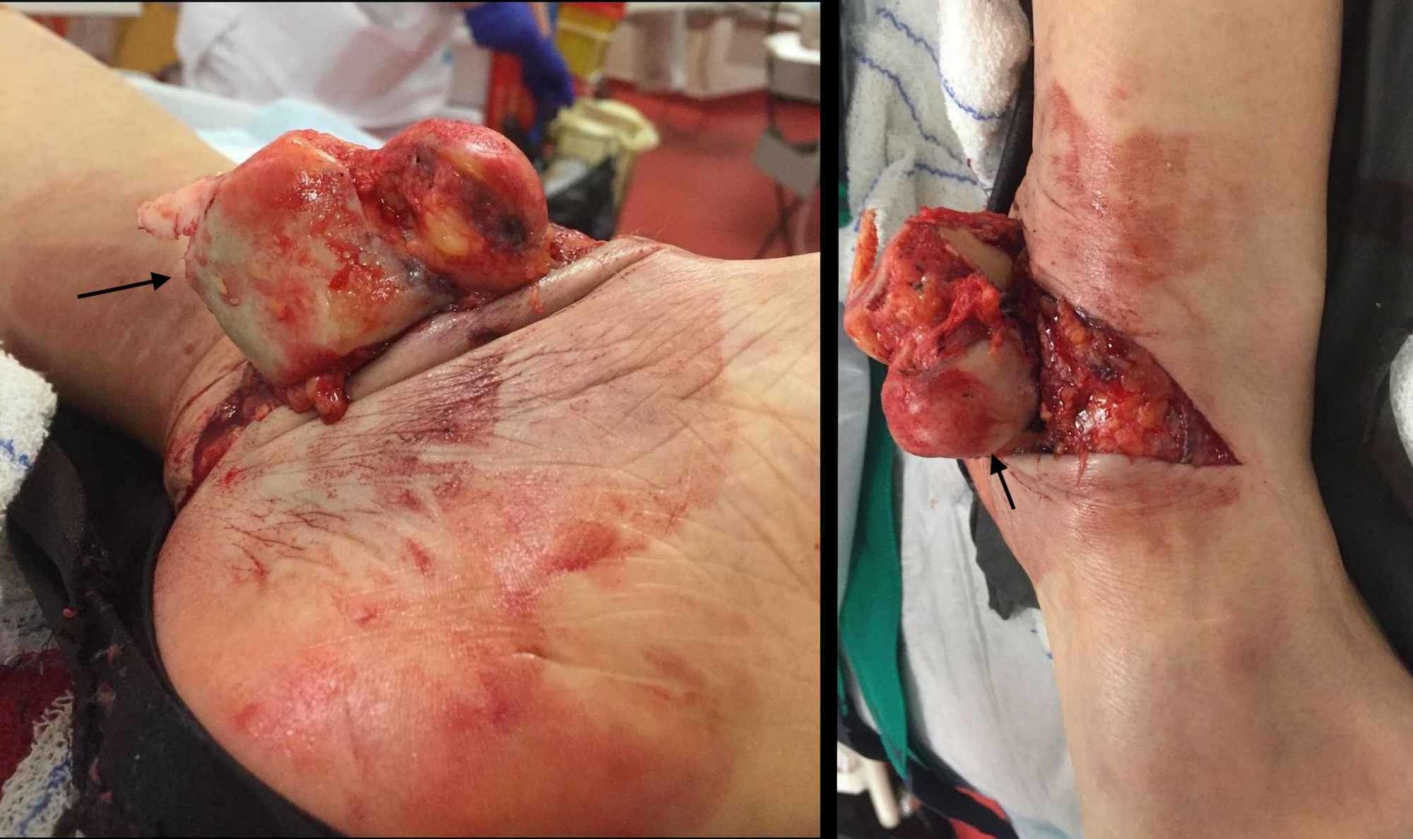
Clinical image showing approximately 12-cm laceration on the medial aspect of the left ankle with a talus totally extruded through the skin

A 67-year-old woman was admitted to the emergency room for an open trauma of the left ankle after a car accident. Initial physical examination showed an approximately 12-cm laceration on the medial aspect of the left ankle with a talus totally extruded through the skin (Figure 1).

**Figure 2:**
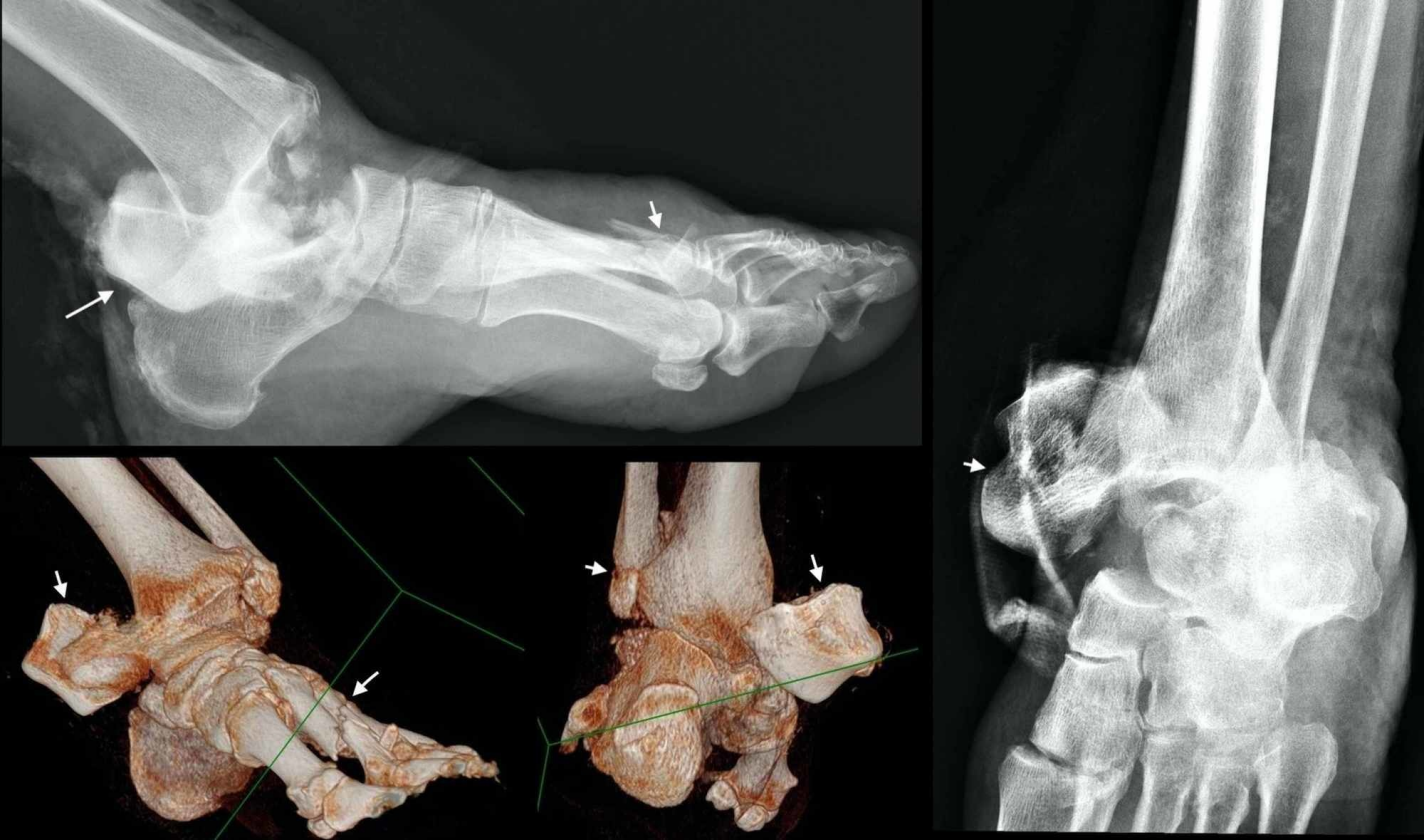
Radiographs and a CT scan of the left ankle showing an extrusion of the talus with multiple fractures (distal fibula, tarsal and metatarsal bones)

Initial radiographs and a CT scan of the left ankle revealed an extrusion of the talus with multiple fractures (distal fibula, tarsal and metatarsal bones) (Figure 2).

**Figure 3:**
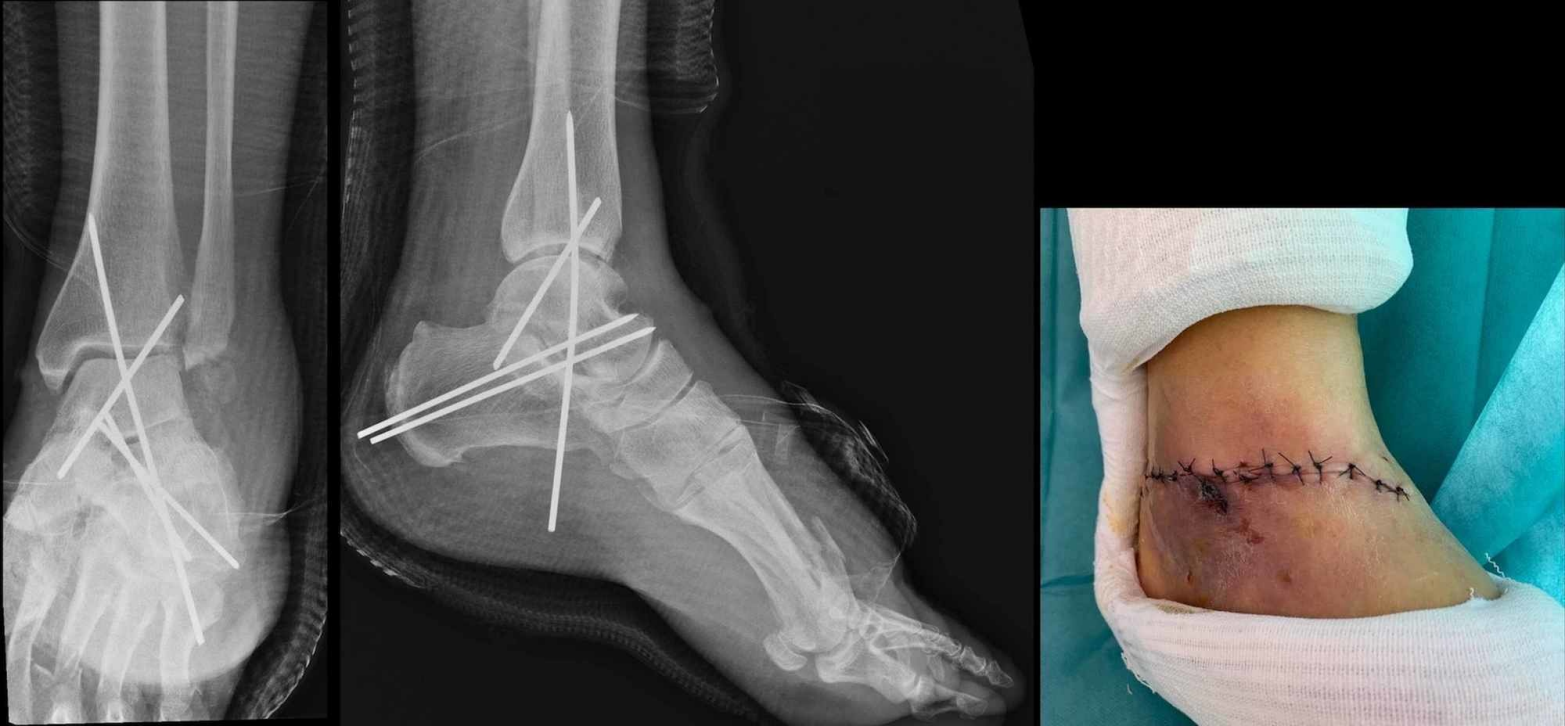
Postoperative radiographs showing a good reduction of the talus and fixation with 4 K-wire (22/10) through the talocrural and subtalar joints

Two grams of amoxicillin-clavulanic acid was administered before surgery with a tetanus toxoid booster. Then, the patient was shifted to the operating room. Wound irrigation and debridement were performed, and the talus was thoroughly cleaned by pulsatile lavage. The final diagnosis was a type 3a Gustilo open fracture of the left ankle with a total talar extrusion. Surgical exploration found a 12-cm wound on the medial aspect of the left ankle. A few attachments on the medial side of the talus were preserved along with the posterior tibial artery and the great saphenous vein. The talus was immediately reimplanted and fixed under fluoroscopy by 4 K-wire (22/10) through the talocrural and subtalar joints (Figure 3).

**Figure 4:**
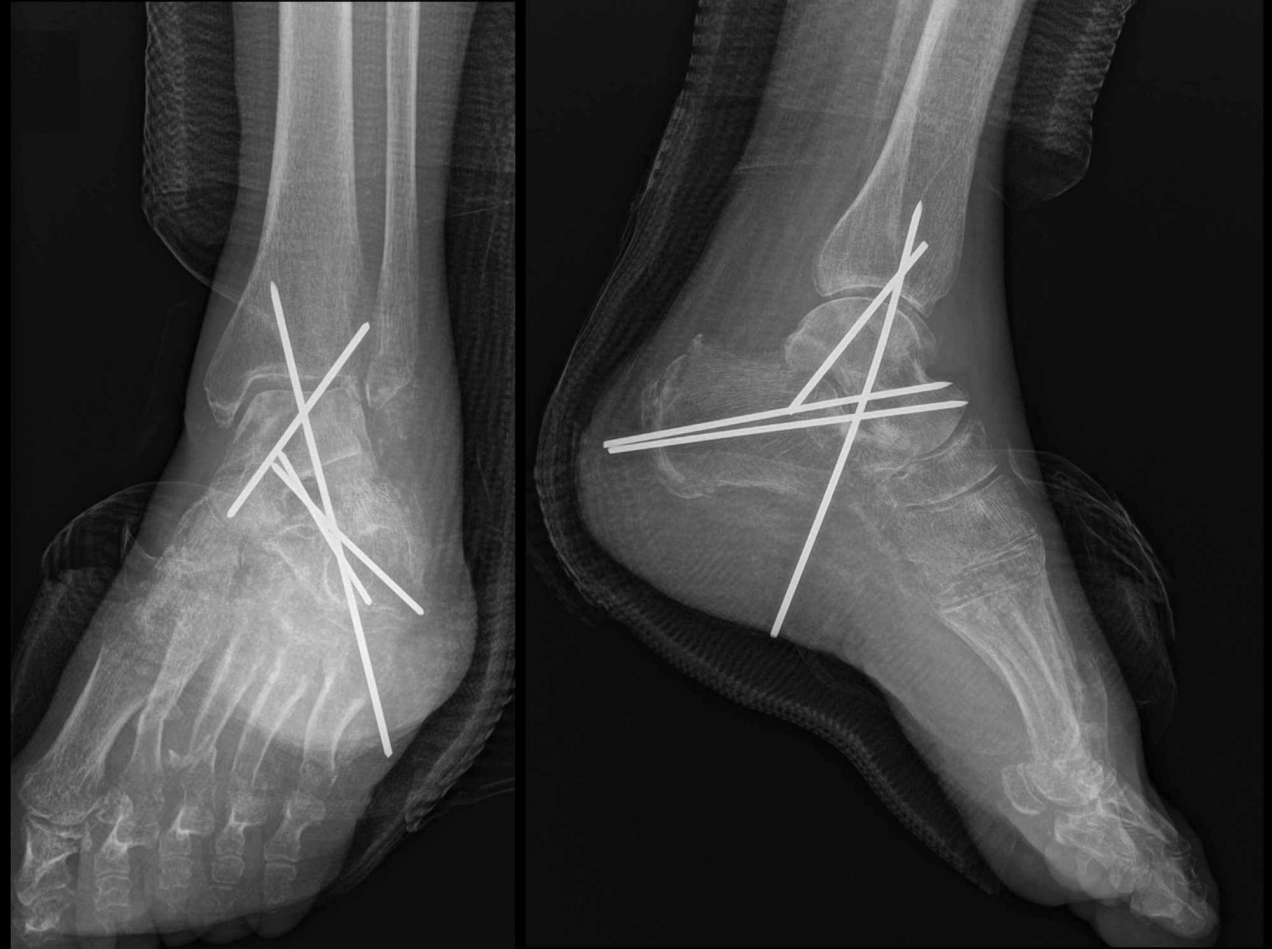
Radiography at three months showing an osteopenia of the ankle joints secondary to immobilization

The ankle was stable intraoperatively; we judged that the lateral malleolus fracture did not need to be fixed and we removed the small bony fragments. After final irrigation with saline and hemostasis, we repaired the medial capsular and ligament tear, and then the wound was closed without tension. A cast was applied with instructions for no weight-bearing for three months alongside painkillers and anti-thrombosis therapy. No complications had occurred through three months of follow-up, and then we removed the K-wires and the cast. The mobilization of the ankle without weight-bearing was allowed. Radiography showed an osteopenia of the ankle joints secondary to immobilization (Figure 4).

**Figure 5:**
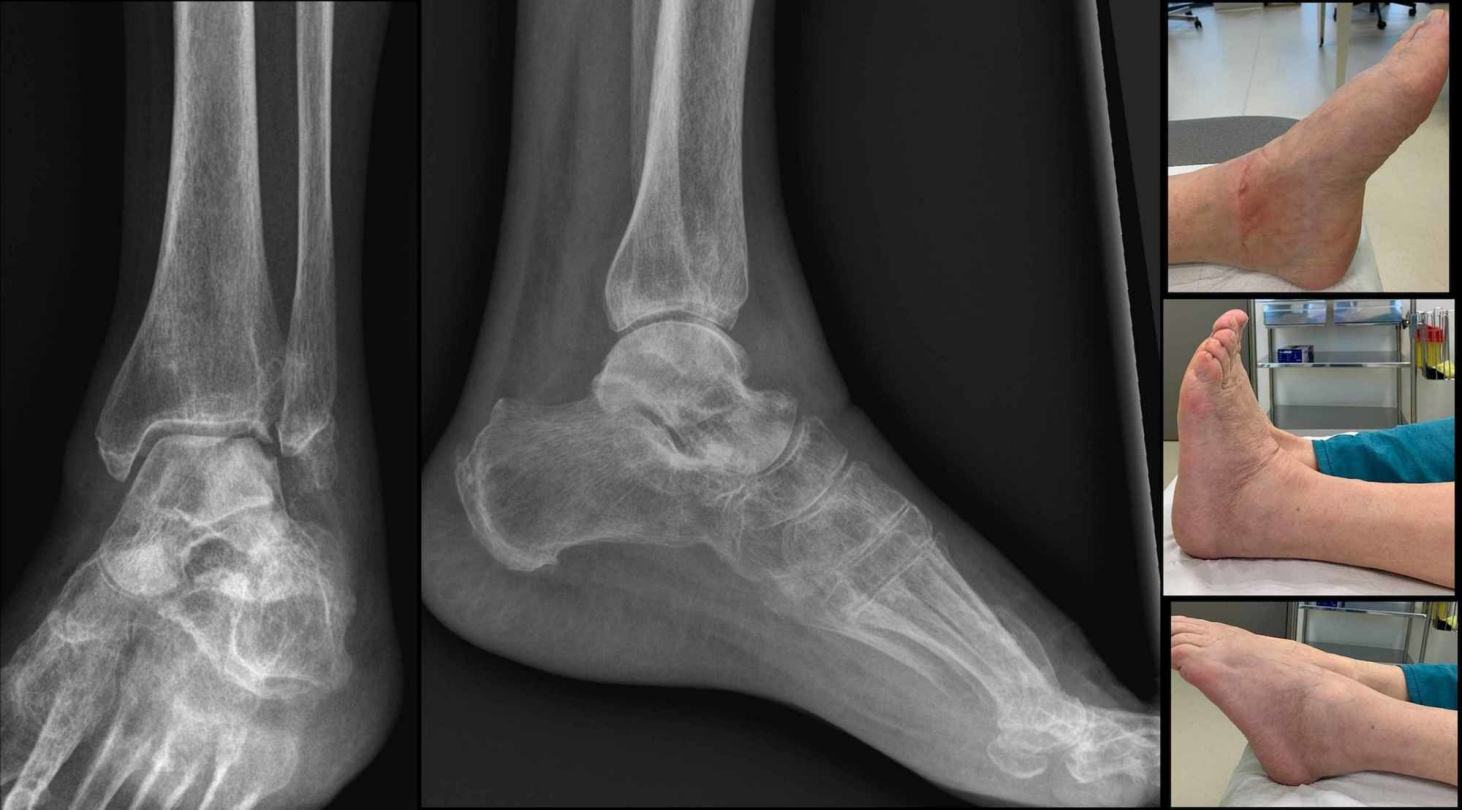
Clinical and radiological outcomes one year after the accident

After one year, the patient was walking with full weight-bearing without aid or pain, and the ankle joint range of motion (ROM) was 40 degrees of plantar flexion and 15 degrees of dorsiflexion (Figure 5).

## Discussion

Total traumatic extrusion of the talus is a rare injury that occurs after a high energy trauma. Exaggerated ankle plantar flexion with extreme subtalar supination causes dislocation and disruption of the ligaments. Many treatment options are described in the literature, but none of them garant a successful result [4]. Assuredly, there is no consensus about appropriate treatment [3,6,7]. Not long ago, talectomy with primary tibiocalcaneal arthrodesis was preferred to reimplantation [8,9]. Palomo-Traver et al. proposed that a totally extruded or grossly contaminated talus should be replaced [10]. Some authors, to reduce the risk of infection, postpone talus reimplantation and use a talus-shaped antibiotic cement while waiting for cultural results [11]. Recent studies with favorable outcomes suggest that immediate reimplantation of the extruded talus is a safest and most appropriate procedure as long as plentiful wound irrigation and debridement are performed to reduce the risk of infection [4,7,10-13]. After reimplantation, fixation can be done with external fixation, Steinmann pins from the calcaneus through the talus into the distal tibia or Kirschner wires [14-17]. In our case, we opted for a more conservative and less invasive treatment considering the early surgical management. Complications of this injury can be separated into short term (infection) and long term (talus collapse, stiffness, arthritis, bone necrosis) [3]. Marsh et al. reported a rate of 38% infection; contrarily, Smith et al. announced a much lower rate [7,18]. In our case, we reduced the risk of infection by following an open fracture protocol: early administration of antibiotics and surgical debridement with a careful soft tissue handling and a rigid fixation [6]. The risk of avascular necrosis of the talus is difficult to predict [19]. It is highest when no soft tissues remain attached to the talus [6]. Hawkins’ sign is the only predictor of talus revascularization and needs to be searched for on conventional radiographs, six to eight weeks from the injury [9]. 

## Conclusions

Open total talar extrusion is a challenging injury. Since there is an absence of guidelines, managing this injury is supported by some cases of class V evidence in the literature. Recently, talus reimplantation after total extrusion became the treatment of choice and showed promising results. In our case, based on the principles of open fracture management, an early administration of antibiotics and tetanus toxoid booster followed by an urgent debridement of the wound and talus primary reimplantation with a lesser invasive K-wire fixation allowed us to achieve a good result.
